# Di­hydro­cyclam dimaleate [H_2_(cyclam)(maleate)_2_]

**DOI:** 10.1107/S1600536813025580

**Published:** 2013-09-21

**Authors:** Mbonzi Ombenga Mireille Ninon, Mohammed Fahim, Mohammed Lachkar, José Luis Marco Contelles, Josefina Perles, Brahim El Bali

**Affiliations:** a LCMSN, Département de Chimie, Faculté des Sciences, Université Moulay Ismail, BP 11201, 50000 Meknès, Morocco; b LIMOM (CNRST, URAC 19), Department of Chemistry, Faculty of Sciences, University Sidi Mohamed Ben Abdellah, BP 1796, 30000 Fès, Morocco; c Laboratorio de Radicales Libres y Quimica Computacional, Instituto de Quimica Organica General, Consejo Superior de Investigaciones Cientificas, C/ Juan de la Cierva, 3, 28006-Madrid, Spain; d Laboratorio de Difracción de Rayos X de Monocristal, Servicio Interdepartamental de Investigación, Universidad Autónoma de Madrid; e LCSMA, Department of Chemistry, Faculty of Sciences, University Mohamed I, Po. Box 717, 60000 Oujda, Morocco

## Abstract

The asymmetric unit of the title mol­ecular salt [systematic name: 1,4,8,11-tetraazacyclotetradecane-1,8-diium bis(3-carboxy­prop-2-enoate)], C_10_H_26_N_4_^2+^·2C_4_H_3_O_4_^−^, contains two half-cations (both completed by crystallographic inversion symmetry) and two maleate anions. The cyclam macrocycles adopt *trans*-III conformations, supported by two intra­molecular N—H⋯O hydrogen bonds. The O-bonded H atom of each maleate ion is disordered over two positions with an occupancy ratio of 0.61 (5):0.39 (5): each one generates an intra­molecular O—H⋯O hydrogen bond. In the crystal, the cations are linked to the anions by N—H⋯O hydrogen bonds, generating [001] chains.

## Related literature

For related cyclam crystal structures, see: Robinson *et al.* (1989) ▶; Frémond *et al.* (2000 ▶); Meyer *et al.* (1998 ▶). For macrocycle conformations, see: Bosnich *et al.* (1965 ▶); Dale (1973 ▶, 1976 ▶); Melson (1979 ▶); Bandoli *et al.* (1993 ▶); Hancock *et al.* (1996 ▶).
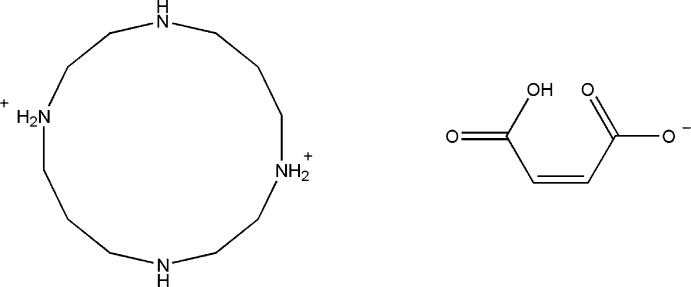


## Experimental

### 

#### Crystal data

C_10_H_26_N_4_^2+^·2C_4_H_3_O_4_^−^
*M* *_r_* = 432.48
Monoclinic, 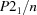

*a* = 8.2925 (1) Å
*b* = 13.5841 (2) Å
*c* = 19.2995 (3) Å
β = 98.797 (1)°
*V* = 2148.44 (5) Å^3^
*Z* = 4
Cu *K*α radiation
μ = 0.89 mm^−1^
*T* = 100 K
0.25 × 0.20 × 0.20 mm

#### Data collection

Bruker SMART CCD diffractometer
Absorption correction: multi-scan (*SADABS*; Bruker, 2005 ▶) *T*_min_ = 0.81, *T*_max_ = 0.84
18854 measured reflections
4018 independent reflections
3821 reflections with *I* > 2σ(*I*)
*R*_int_ = 0.024

#### Refinement

*R*[*F* ^2^ > 2σ(*F* ^2^)] = 0.032
*wR*(*F* ^2^) = 0.083
*S* = 1.04
4018 reflections
403 parameters
H atoms treated by a mixture of independent and constrained refinement
Δρ_max_ = 0.36 e Å^−3^
Δρ_min_ = −0.18 e Å^−3^

### 

Data collection: *SMART* (Bruker, 2005 ▶); cell refinement: *SAINT* (Bruker, 2005 ▶); data reduction: *SAINT*; program(s) used to solve structure: *SHELXS97* (Sheldrick, 2008 ▶); program(s) used to refine structure: *SHELXL97* (Sheldrick, 2008 ▶); molecular graphics: *SHELXTL* (Sheldrick, 2008 ▶); software used to prepare material for publication: *SHELXL97*.

## Supplementary Material

Crystal structure: contains datablock(s) global, I. DOI: 10.1107/S1600536813025580/hb7135sup1.cif

Structure factors: contains datablock(s) I. DOI: 10.1107/S1600536813025580/hb7135Isup2.hkl

Additional supplementary materials:  crystallographic information; 3D view; checkCIF report

## Figures and Tables

**Table 1:** Hydrogen-bond geometry (Å, °)

*D*—H⋯*A*	*D*—H	H⋯*A*	*D*⋯*A*	*D*—H⋯*A*
N1—H1*A*⋯O2^i^	0.920 (17)	1.800 (17)	2.7074 (13)	168.1 (14)
N1—H1*B*⋯N2	0.925 (16)	2.015 (15)	2.8000 (13)	141.7 (13)
N2—H2*A*⋯O7^ii^	0.926 (16)	2.356 (16)	3.2134 (13)	153.9 (13)
N2—H2*A*⋯O8^ii^	0.926 (16)	2.379 (16)	3.2178 (14)	150.6 (13)
N3—H3*A*⋯N4^iii^	0.899 (16)	2.089 (16)	2.8046 (14)	135.8 (13)
N3—H3*B*⋯O5^iv^	0.901 (16)	2.397 (16)	3.0713 (13)	131.7 (12)
N3—H3*B*⋯O6^iv^	0.901 (16)	2.037 (16)	2.8982 (13)	159.5 (14)
N4—H4⋯O4^v^	0.868 (16)	2.348 (16)	3.1596 (12)	155.8 (13)
O3—H3*O*⋯O1	0.90 (3)	1.55 (3)	2.4444 (12)	178 (2)
O7—H7*O*⋯O5	0.92 (4)	1.50 (4)	2.4157 (13)	176 (3)

Symmetry codes: (i) 
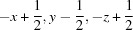
; (ii) 
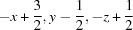
; (iii) 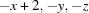; (iv) 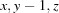; (v) 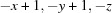.

**Fig. 1.**
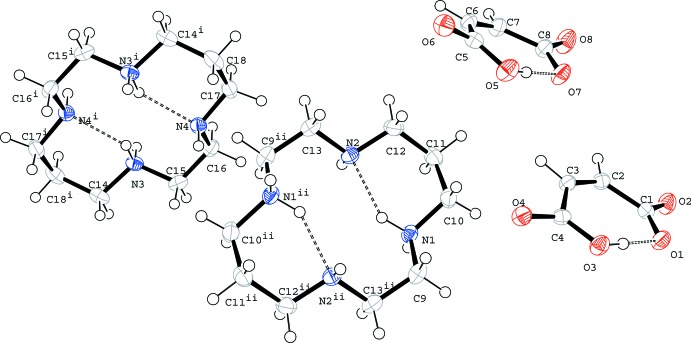
: The molecular structure of the title compound, with hydrogen bonds shown as dashed lines. Anisotropic displacement parameters drawn at the 50% probability level.

**Fig. 2.**
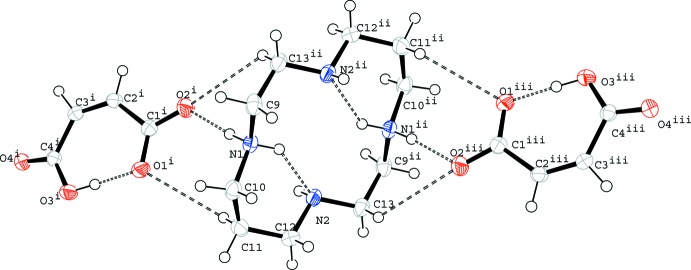
: Inter-molecular H-bonds between cyclam and maleate ion.

**Fig. 3.**
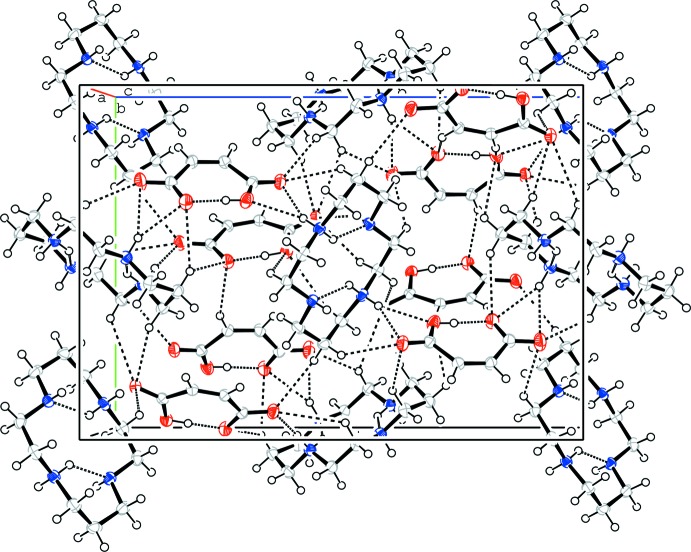
: Projection of the structure in bc-plane, hydrogen bonds as dashed lines.

## supplementary crystallographic information

### 1. Comment

Cyclam is doubly protonated in [H_2_(cyclam)(maleate)_2_], resulting in a maleate monoanion. The di-protonated cyclam [C_10_H_26_N_4_]^2+^ exhibits bond distances and angles in the range usually found in the literature (Melson, 1979). The two additional protons on N1 and N1A are *trans* to each other and interact through hydrogen bonds with the nonprotonated N2 and N2A nitrogen atoms [N1···N2= 2.800 (1) Å, N3···N4= 2.805 (1) Å]. The diprotonated macrocycle adopts an quadrangular (3,4,3,4)-C conformation (Fig. 1) according to Dale's nomenclature [(Dale, 1973 and 1976, (Hancock *et al.*, 1996)], where the *exo*-cyclic nitrogen atom N2 is located two bonds away from the adjacent corner atoms C9A and C11, while the amine nitrogen atom N1A is located one bond away from the corresponding corner atom C9A and two bonds away from C11A. According to the stereochemical classification of 1,4,8,11 tetraazacyclotetradecane introduced by Bosnich *et al.* (1965), the cyclam ring adopts a *trans*-III geometry type, which, according to molecular mechanics *MM*-calculations, is the most stable among the five possible configurations (Bandoli *et al.*, 1993). In the crystal structure, intramolecular hydrogen bonds occur, linking carboxylate O atoms in each maleate ion. The H3C and H7A atoms are involved in these bonds and maintain the charge balance by bridging two carboxylate groups within the structure. The complex features intra and intermolecular hydrogen bonds involving N—H···O, N—H···N and O—H···O. The main types of such bonds are those between protonated NH groups of the marcocycle and O atoms of the ionized maleic hydroxyls, between protonated and nonprotonated NH groups of the macrocycle and between O-atoms of the same ionized maleic hydroxyls as shown on Fig. 2 and 3. The values of the main H-bonds are reported in table 3.

### 2. Experimental

0.2 g (1 mmol) of cyclam C_10_H_24_N_4_ was dissolved 25 ml of water-ethanol mixture 1:1 (*v*/*v*) and 0.233 g (2 mmol) of maleic acid C_4_H_4_O_4_, in 25 ml of the same solvent. The solutions were combined, and the mixture was refluxed for 3 h, before to be deposited to set at room conditions. Prismatic coloreless crystals were obtained which were washed with a water (80%) /ethanol (20%) (v:v) solution.

### Crystal data

**Table:**

C_10_H_26_N_4_^2+^·2C_4_H_3_O_4_^−^	*Z* = 4
*M**_r_* = 432.48	*F*(000) = 928
Monoclinic, *P*2_1_/*n*	*D*_x_ = 1.337 Mg m^−^^3^
*a* = 8.2925 (1) Å	Cu *K*α radiation, λ = 1.54178 Å
*b* = 13.5841 (2) Å	µ = 0.89 mm^−^^1^
*c* = 19.2995 (3) Å	*T* = 100 K
β = 98.797 (1)°	Prism, colourless
*V* = 2148.44 (5) Å^3^	0.25 × 0.20 × 0.20 mm

### Data collection

**Table:**

Bruker SMART CCD diffractometer	3821 reflections with *I* > 2σ(*I*)
ω scans	*R*_int_ = 0.024
Absorption correction: multi-scan (*SADABS*; Bruker, 2005)	θ_max_ = 70.1°, θ_min_ = 4.6°
*T*_min_ = 0.81, *T*_max_ = 0.84	*h* = −10→9
18854 measured reflections	*k* = −16→16
4018 independent reflections	*l* = −23→23

### Refinement

**Table:**

Refinement on *F*^2^	Primary atom site location: structure-invariant direct methods
Least-squares matrix: full	Secondary atom site location: difference Fourier map
*R*[*F*^2^ > 2σ(*F*^2^)] = 0.032	Hydrogen site location: inferred from neighbouring sites
*wR*(*F*^2^) = 0.083	H atoms treated by a mixture of independent and constrained refinement
*S* = 1.04	*w* = 1/[σ^2^(*F*_o_^2^) + (0.0406*P*)^2^ + 0.6721*P*] where *P* = (*F*_o_^2^ + 2*F*_c_^2^)/3
4018 reflections	(Δ/σ)_max_ < 0.001
403 parameters	Δρ_max_ = 0.36 e Å^−^^3^
0 restraints	Δρ_min_ = −0.18 e Å^−^^3^

### Special details

**Table:**

Geometry. All e.s.d.'s (except the e.s.d. in the dihedral angle between two l.s. planes) are estimated using the full covariance matrix. The cell e.s.d.'s are taken into account individually in the estimation of e.s.d.'s in distances, angles and torsion angles; correlations between e.s.d.'s in cell parameters are only used when they are defined by crystal symmetry. An approximate (isotropic) treatment of cell e.s.d.'s is used for estimating e.s.d.'s involving l.s. planes.
Refinement. Refinement of *F*^2^ against ALL reflections. The weighted *R*-factor *wR* and goodness of fit *S* are based on *F*^2^, conventional *R*-factors *R* are based on *F*, with *F* set to zero for negative *F*^2^. The threshold expression of *F*^2^ > σ(*F*^2^) is used only for calculating *R*-factors(gt) *etc*. and is not relevant to the choice of reflections for refinement. *R*-factors based on *F*^2^ are statistically about twice as large as those based on *F*, and *R*- factors based on ALL data will be even larger.

### Fractional atomic coordinates and isotropic or equivalent isotropic displacement parameters (Å^2^)

**Table:**

	*x*	*y*	*z*	*U*_iso_*/*U*_eq_	Occ. (<1)
C1	0.29130 (13)	0.94686 (8)	0.28537 (6)	0.0241 (2)	
C2	0.42219 (13)	0.89074 (8)	0.25661 (6)	0.0240 (2)	
C3	0.44193 (13)	0.87391 (8)	0.19031 (6)	0.0229 (2)	
C4	0.34069 (12)	0.90322 (8)	0.12233 (6)	0.0227 (2)	
C5	0.98341 (14)	0.76765 (8)	0.18188 (6)	0.0260 (2)	
C6	1.05748 (14)	0.70568 (9)	0.24264 (6)	0.0259 (2)	
C7	1.02908 (14)	0.70577 (9)	0.30874 (6)	0.0274 (2)	
C8	0.92026 (14)	0.76946 (9)	0.34489 (6)	0.0284 (2)	
C9	0.25226 (15)	0.61005 (9)	0.03030 (6)	0.0301 (3)	
C10	0.47726 (15)	0.63339 (9)	0.13169 (7)	0.0303 (3)	
C11	0.62753 (15)	0.58461 (10)	0.17098 (6)	0.0319 (3)	
C12	0.75542 (14)	0.55991 (10)	0.12458 (7)	0.0309 (3)	
C13	0.81528 (14)	0.46284 (9)	0.02577 (6)	0.0289 (3)	
C14	0.75571 (15)	−0.17204 (9)	−0.00831 (7)	0.0313 (3)	
C15	0.74127 (14)	−0.02422 (9)	0.06666 (6)	0.0283 (2)	
C16	0.84068 (14)	0.05815 (9)	0.10416 (6)	0.0281 (2)	
C17	1.02932 (15)	0.19107 (9)	0.08931 (6)	0.0306 (3)	
C18	1.12976 (16)	0.24033 (9)	0.03941 (7)	0.0335 (3)	
N1	0.38474 (12)	0.56356 (7)	0.08060 (5)	0.0245 (2)	
N2	0.69367 (11)	0.48659 (7)	0.07078 (5)	0.0258 (2)	
N3	0.84805 (11)	−0.09675 (7)	0.03828 (5)	0.0243 (2)	
N4	0.92438 (12)	0.11249 (7)	0.05474 (5)	0.0248 (2)	
O1	0.18816 (10)	0.99727 (6)	0.24442 (4)	0.03003 (19)	
H1O	0.2061	0.9906	0.203	0.045*	0.39 (5)
O2	0.29456 (10)	0.94215 (6)	0.35007 (4)	0.0326 (2)	
O3	0.22552 (10)	0.96878 (6)	0.12282 (4)	0.02853 (19)	
O4	0.37276 (9)	0.86677 (6)	0.06808 (4)	0.02820 (19)	
O5	0.85713 (11)	0.82014 (7)	0.18682 (5)	0.0352 (2)	
H5O	0.8402	0.8206	0.2286	0.053*	0.41 (5)
O6	1.04578 (11)	0.76384 (7)	0.12791 (4)	0.0332 (2)	
O7	0.81387 (10)	0.82564 (7)	0.30773 (5)	0.0340 (2)	
O8	0.93463 (12)	0.76651 (8)	0.40874 (5)	0.0430 (2)	
H1A	0.3368 (19)	0.5175 (12)	0.1059 (8)	0.036 (4)*	
H1B	0.4603 (19)	0.5331 (11)	0.0571 (8)	0.034 (4)*	
H2	0.4994 (17)	0.8649 (10)	0.2919 (7)	0.026 (3)*	
H2A	0.6715 (18)	0.4294 (12)	0.0937 (8)	0.035 (4)*	
H3	0.5346 (17)	0.8353 (10)	0.1825 (7)	0.026 (3)*	
H3A	0.9173 (18)	−0.0670 (11)	0.0134 (8)	0.032 (4)*	
H3B	0.9109 (19)	−0.1278 (11)	0.0739 (8)	0.034 (4)*	
H3O	0.211 (3)	0.9802 (17)	0.1673 (15)	0.023 (8)*	0.61 (5)
H4	0.8494 (19)	0.1373 (11)	0.0233 (8)	0.033 (4)*	
H6	1.1379 (17)	0.6605 (11)	0.2304 (8)	0.031 (3)*	
H7	1.0899 (18)	0.6622 (12)	0.3405 (8)	0.035 (4)*	
H7O	0.829 (3)	0.826 (2)	0.2615 (19)	0.033 (10)*	0.59 (5)
H9A	0.3008 (18)	0.6660 (12)	0.0107 (8)	0.036 (4)*	
H9B	0.1683 (17)	0.6323 (11)	0.0580 (8)	0.031 (3)*	
H10A	0.5089 (18)	0.6906 (11)	0.1055 (8)	0.033 (4)*	
H10B	0.4040 (17)	0.6548 (11)	0.1624 (8)	0.031 (3)*	
H11A	0.6791 (19)	0.6312 (12)	0.2085 (8)	0.039 (4)*	
H11B	0.5941 (17)	0.5260 (11)	0.1945 (8)	0.031 (3)*	
H12A	0.7842 (17)	0.6192 (11)	0.0991 (7)	0.030 (3)*	
H12B	0.855 (2)	0.5371 (11)	0.1536 (8)	0.038 (4)*	
H13A	0.8453 (17)	0.5260 (11)	0.0028 (7)	0.028 (3)*	
H13B	0.9123 (19)	0.4366 (11)	0.0525 (8)	0.033 (4)*	
H14A	0.6835 (18)	−0.1343 (11)	−0.0437 (8)	0.034 (4)*	
H14B	0.6853 (19)	−0.2065 (12)	0.0195 (8)	0.039 (4)*	
H15A	0.6656 (17)	0.0007 (10)	0.0272 (7)	0.025 (3)*	
H15B	0.6817 (18)	−0.0594 (11)	0.0973 (8)	0.034 (4)*	
H16A	0.9219 (17)	0.0315 (10)	0.1411 (7)	0.028 (3)*	
H16B	0.7675 (18)	0.1014 (11)	0.1274 (8)	0.033 (4)*	
H17A	1.1036 (17)	0.1611 (10)	0.1290 (7)	0.028 (3)*	
H17B	0.9618 (18)	0.2419 (12)	0.1109 (8)	0.036 (4)*	
H18A	1.0582 (19)	0.2722 (11)	0.0018 (8)	0.036 (4)*	
H18B	1.1958 (19)	0.2909 (12)	0.0640 (8)	0.039 (4)*	

### Atomic displacement parameters (Å^2^)

**Table:**

	*U*^11^	*U*^22^	*U*^33^	*U*^12^	*U*^13^	*U*^23^
C1	0.0230 (5)	0.0242 (5)	0.0252 (5)	−0.0015 (4)	0.0038 (4)	−0.0028 (4)
C2	0.0228 (5)	0.0247 (5)	0.0235 (5)	0.0025 (4)	0.0007 (4)	0.0005 (4)
C3	0.0196 (5)	0.0231 (5)	0.0261 (5)	0.0023 (4)	0.0034 (4)	0.0001 (4)
C4	0.0201 (5)	0.0239 (5)	0.0243 (5)	−0.0021 (4)	0.0040 (4)	0.0022 (4)
C5	0.0261 (5)	0.0270 (5)	0.0238 (5)	−0.0043 (4)	0.0005 (4)	0.0003 (4)
C6	0.0235 (5)	0.0275 (6)	0.0261 (6)	0.0040 (4)	0.0025 (4)	−0.0013 (4)
C7	0.0267 (6)	0.0300 (6)	0.0245 (5)	0.0073 (5)	0.0006 (4)	0.0031 (5)
C8	0.0265 (6)	0.0319 (6)	0.0270 (6)	0.0035 (5)	0.0046 (4)	−0.0003 (5)
C9	0.0322 (6)	0.0314 (6)	0.0286 (6)	0.0088 (5)	0.0108 (5)	0.0045 (5)
C10	0.0332 (6)	0.0265 (6)	0.0337 (6)	−0.0034 (5)	0.0133 (5)	−0.0063 (5)
C11	0.0350 (6)	0.0339 (6)	0.0273 (6)	−0.0077 (5)	0.0066 (5)	−0.0046 (5)
C12	0.0243 (6)	0.0366 (6)	0.0312 (6)	−0.0054 (5)	0.0029 (5)	−0.0024 (5)
C13	0.0234 (5)	0.0362 (6)	0.0283 (6)	0.0052 (5)	0.0072 (5)	0.0056 (5)
C14	0.0302 (6)	0.0323 (6)	0.0291 (6)	−0.0103 (5)	−0.0024 (5)	0.0020 (5)
C15	0.0209 (5)	0.0360 (6)	0.0283 (6)	0.0020 (5)	0.0051 (4)	0.0075 (5)
C16	0.0259 (6)	0.0359 (6)	0.0230 (5)	0.0077 (5)	0.0056 (4)	−0.0002 (5)
C17	0.0331 (6)	0.0273 (6)	0.0290 (6)	0.0034 (5)	−0.0030 (5)	−0.0068 (5)
C18	0.0398 (7)	0.0236 (6)	0.0328 (6)	−0.0046 (5)	−0.0083 (5)	−0.0007 (5)
N1	0.0243 (5)	0.0245 (5)	0.0265 (5)	0.0006 (4)	0.0098 (4)	−0.0007 (4)
N2	0.0234 (5)	0.0284 (5)	0.0268 (5)	−0.0021 (4)	0.0082 (4)	0.0003 (4)
N3	0.0224 (5)	0.0269 (5)	0.0229 (5)	−0.0033 (4)	0.0007 (4)	0.0039 (4)
N4	0.0246 (5)	0.0258 (5)	0.0227 (5)	0.0031 (4)	−0.0009 (4)	−0.0007 (4)
O1	0.0262 (4)	0.0355 (4)	0.0282 (4)	0.0088 (3)	0.0038 (3)	−0.0013 (4)
O2	0.0365 (5)	0.0374 (5)	0.0247 (4)	0.0074 (4)	0.0076 (3)	−0.0025 (3)
O3	0.0278 (4)	0.0313 (4)	0.0257 (4)	0.0082 (3)	0.0017 (3)	0.0031 (3)
O4	0.0275 (4)	0.0357 (4)	0.0216 (4)	0.0025 (3)	0.0045 (3)	0.0008 (3)
O5	0.0337 (5)	0.0425 (5)	0.0286 (5)	0.0118 (4)	0.0026 (3)	0.0092 (4)
O6	0.0375 (5)	0.0395 (5)	0.0232 (4)	−0.0024 (4)	0.0064 (3)	0.0016 (3)
O7	0.0311 (4)	0.0404 (5)	0.0306 (5)	0.0142 (4)	0.0053 (3)	0.0018 (4)
O8	0.0473 (5)	0.0572 (6)	0.0254 (4)	0.0175 (5)	0.0083 (4)	−0.0004 (4)

### Geometric parameters (Å, º)

**Table:**

C1—O2	1.2463 (14)	C13—N2	1.4641 (14)
C1—O1	1.2722 (14)	C13—C9^i^	1.5099 (18)
C1—C2	1.5007 (15)	C13—H13A	1.014 (15)
C2—C3	1.3343 (16)	C13—H13B	0.956 (16)
C2—H2	0.930 (14)	C14—N3	1.4933 (15)
C3—C4	1.4991 (15)	C14—C18^ii^	1.5156 (19)
C3—H3	0.961 (14)	C14—H14A	0.981 (16)
C4—O4	1.2236 (14)	C14—H14B	0.972 (16)
C4—O3	1.3070 (14)	C15—N3	1.4843 (15)
C5—O6	1.2328 (14)	C15—C16	1.5076 (17)
C5—O5	1.2824 (15)	C15—H15A	0.971 (14)
C5—C6	1.4969 (16)	C15—H15B	0.954 (16)
C6—C7	1.3321 (17)	C16—N4	1.4625 (15)
C6—H6	0.962 (15)	C16—H16A	0.973 (14)
C7—C8	1.4975 (16)	C16—H16B	0.998 (15)
C7—H7	0.941 (16)	C17—N4	1.4712 (15)
C8—O8	1.2204 (15)	C17—C18	1.5213 (19)
C8—O7	1.2967 (15)	C17—H17A	0.994 (14)
C9—N1	1.4906 (15)	C17—H17B	1.017 (16)
C9—C13^i^	1.5099 (18)	C18—C14^ii^	1.5155 (19)
C9—H9A	0.963 (16)	C18—H18A	0.967 (16)
C9—H9B	0.988 (15)	C18—H18B	0.958 (17)
C10—N1	1.4929 (15)	N1—H1A	0.920 (17)
C10—C11	1.5093 (18)	N1—H1B	0.925 (16)
C10—H10A	0.984 (16)	N2—H2A	0.926 (16)
C10—H10B	0.956 (15)	N3—H3A	0.899 (16)
C11—C12	1.5262 (17)	N3—H3B	0.901 (16)
C11—H11A	1.007 (16)	N4—H4	0.868 (16)
C11—H11B	0.978 (15)	O1—H1O	0.84
C12—N2	1.4729 (16)	O3—H3O	0.90 (3)
C12—H12A	0.992 (15)	O5—H5O	0.84
C12—H12B	0.977 (16)	O7—H7O	0.92 (4)
			
O2—C1—O1	124.03 (10)	H13A—C13—H13B	108.0 (12)
O2—C1—C2	116.00 (10)	N3—C14—C18^ii^	111.28 (10)
O1—C1—C2	119.95 (10)	N3—C14—H14A	105.3 (9)
C3—C2—C1	130.06 (10)	C18^ii^—C14—H14A	113.4 (9)
C3—C2—H2	117.8 (8)	N3—C14—H14B	107.1 (9)
C1—C2—H2	112.1 (8)	C18^ii^—C14—H14B	112.9 (9)
C2—C3—C4	131.25 (10)	H14A—C14—H14B	106.4 (12)
C2—C3—H3	117.6 (8)	N3—C15—C16	110.89 (9)
C4—C3—H3	111.2 (8)	N3—C15—H15A	107.0 (8)
O4—C4—O3	122.45 (10)	C16—C15—H15A	111.0 (8)
O4—C4—C3	118.39 (10)	N3—C15—H15B	106.9 (9)
O3—C4—C3	119.12 (9)	C16—C15—H15B	111.8 (9)
O6—C5—O5	122.62 (11)	H15A—C15—H15B	109.1 (12)
O6—C5—C6	117.60 (10)	N4—C16—C15	109.92 (9)
O5—C5—C6	119.75 (10)	N4—C16—H16A	108.8 (8)
C7—C6—C5	129.66 (11)	C15—C16—H16A	110.0 (8)
C7—C6—H6	117.9 (9)	N4—C16—H16B	112.1 (8)
C5—C6—H6	112.5 (9)	C15—C16—H16B	109.1 (8)
C6—C7—C8	130.85 (11)	H16A—C16—H16B	106.9 (12)
C6—C7—H7	117.9 (9)	N4—C17—C18	112.03 (10)
C8—C7—H7	111.1 (9)	N4—C17—H17A	107.8 (8)
O8—C8—O7	122.06 (11)	C18—C17—H17A	109.5 (8)
O8—C8—C7	118.61 (11)	N4—C17—H17B	110.8 (8)
O7—C8—C7	119.33 (10)	C18—C17—H17B	110.4 (9)
N1—C9—C13^i^	110.16 (9)	H17A—C17—H17B	106.1 (12)
N1—C9—H9A	106.2 (9)	C14^ii^—C18—C17	114.74 (10)
C13^i^—C9—H9A	111.6 (9)	C14^ii^—C18—H18A	109.1 (9)
N1—C9—H9B	106.7 (8)	C17—C18—H18A	109.9 (9)
C13^i^—C9—H9B	111.9 (8)	C14^ii^—C18—H18B	106.9 (9)
H9A—C9—H9B	110.0 (12)	C17—C18—H18B	109.1 (9)
N1—C10—C11	110.75 (10)	H18A—C18—H18B	106.8 (13)
N1—C10—H10A	108.3 (9)	C9—N1—C10	114.46 (9)
C11—C10—H10A	109.9 (9)	C9—N1—H1A	107.3 (10)
N1—C10—H10B	106.9 (9)	C10—N1—H1A	107.6 (10)
C11—C10—H10B	112.1 (9)	C9—N1—H1B	110.9 (9)
H10A—C10—H10B	108.7 (12)	C10—N1—H1B	106.8 (9)
C10—C11—C12	113.34 (10)	H1A—N1—H1B	109.6 (13)
C10—C11—H11A	108.2 (9)	C13—N2—C12	111.75 (9)
C12—C11—H11A	107.7 (9)	C13—N2—H2A	107.7 (9)
C10—C11—H11B	108.7 (8)	C12—N2—H2A	107.7 (9)
C12—C11—H11B	111.5 (8)	C15—N3—C14	113.39 (9)
H11A—C11—H11B	107.3 (12)	C15—N3—H3A	111.3 (9)
N2—C12—C11	111.37 (10)	C14—N3—H3A	107.3 (9)
N2—C12—H12A	106.5 (8)	C15—N3—H3B	109.7 (10)
C11—C12—H12A	110.7 (8)	C14—N3—H3B	108.8 (10)
N2—C12—H12B	111.2 (9)	H3A—N3—H3B	106.0 (13)
C11—C12—H12B	109.8 (9)	C16—N4—C17	112.16 (9)
H12A—C12—H12B	107.1 (12)	C16—N4—H4	106.9 (10)
N2—C13—C9^i^	110.86 (10)	C17—N4—H4	110.3 (10)
N2—C13—H13A	108.1 (8)	C1—O1—H1O	109.5
C9^i^—C13—H13A	109.3 (8)	C4—O3—H3O	109.3 (14)
N2—C13—H13B	111.2 (9)	C5—O5—H5O	109.5
C9^i^—C13—H13B	109.4 (9)	C8—O7—H7O	111.2 (16)
			
O2—C1—C2—C3	172.92 (12)	C10—C11—C12—N2	−64.14 (14)
O1—C1—C2—C3	−8.76 (18)	N3—C15—C16—N4	63.16 (12)
C1—C2—C3—C4	−1.1 (2)	N4—C17—C18—C14^ii^	−60.25 (13)
C2—C3—C4—O4	−169.79 (12)	C13^i^—C9—N1—C10	171.04 (9)
C2—C3—C4—O3	12.43 (18)	C11—C10—N1—C9	−169.21 (10)
O6—C5—C6—C7	169.97 (12)	C9^i^—C13—N2—C12	−178.28 (10)
O5—C5—C6—C7	−11.73 (19)	C11—C12—N2—C13	179.69 (10)
C5—C6—C7—C8	−2.7 (2)	C16—C15—N3—C14	−171.53 (10)
C6—C7—C8—O8	−167.94 (13)	C18^ii^—C14—N3—C15	176.49 (10)
C6—C7—C8—O7	12.2 (2)	C15—C16—N4—C17	−177.39 (9)
N1—C10—C11—C12	67.08 (13)	C18—C17—N4—C16	173.98 (10)

Symmetry codes: (i) −*x*+1, −*y*+1, −*z*; (ii) −*x*+2, −*y*, −*z*.

### Hydrogen-bond geometry (Å, º)

**Table:**

*D*—H···*A*	*D*—H	H···*A*	*D*···*A*	*D*—H···*A*
N1—H1*A*···O2^iii^	0.920 (17)	1.800 (17)	2.7074 (13)	168.1 (14)
N1—H1*B*···N2	0.925 (16)	2.015 (15)	2.8000 (13)	141.7 (13)
N2—H2*A*···O7^iv^	0.926 (16)	2.356 (16)	3.2134 (13)	153.9 (13)
N2—H2*A*···O8^iv^	0.926 (16)	2.379 (16)	3.2178 (14)	150.6 (13)
N3—H3*A*···N4^ii^	0.899 (16)	2.089 (16)	2.8046 (14)	135.8 (13)
N3—H3*B*···O5^v^	0.901 (16)	2.397 (16)	3.0713 (13)	131.7 (12)
N3—H3*B*···O6^v^	0.901 (16)	2.037 (16)	2.8982 (13)	159.5 (14)
N4—H4···O4^i^	0.868 (16)	2.348 (16)	3.1596 (12)	155.8 (13)
O3—H3*O*···O1	0.90 (3)	1.55 (3)	2.4444 (12)	178 (2)
O7—H7*O*···O5	0.92 (4)	1.50 (4)	2.4157 (13)	176 (3)

Symmetry codes: (i) −*x*+1, −*y*+1, −*z*; (ii) −*x*+2, −*y*, −*z*; (iii) −*x*+1/2, *y*−1/2, −*z*+1/2; (iv) −*x*+3/2, *y*−1/2, −*z*+1/2; (v) *x*, *y*−1, *z*.
